# The Lethal Fungus *Batrachochytrium dendrobatidis* Is Present in Lowland Tropical Forests of Far Eastern Panamá

**DOI:** 10.1371/journal.pone.0095484

**Published:** 2014-04-16

**Authors:** Eria A. Rebollar, Myra C. Hughey, Reid N. Harris, Rickie J. Domangue, Daniel Medina, Roberto Ibáñez, Lisa K. Belden

**Affiliations:** 1 Department of Biology, James Madison University, Harrisonburg, Virginia, United States of America; 2 Department of Biological Sciences, Virginia Tech, Blacksburg, Virginia, United States of America; 3 Department of Mathematics and Statistics, James Madison University, Harrisonburg, Virginia, United States of America; 4 Smithsonian Tropical Research Institute, Balboa, Ancón, Republic of Panamá; Leibniz Institute for Natural Products Research and Infection Biology- Hans Knoell Institute, Germany

## Abstract

The fungal disease chytridiomycosis, caused by *Batrachochytrium dendrobatidis* (*Bd*), is one of the main causes of amphibian population declines and extinctions all over the world. In the Neotropics, this fungal disease has caused catastrophic declines in the highlands as it has spread throughout Central America down to Panamá. In this study, we determined the prevalence and intensity of *Bd* infection in three species of frogs in one highland and four lowland tropical forests, including two lowland regions in eastern Panamá in which the pathogen had not been detected previously. *Bd* was present in all the sites sampled with a prevalence ranging from 15–34%, similar to other Neotropical lowland sites. The intensity of *Bd* infection on individual frogs was low, ranging from average values of 0.11–24 zoospore equivalents per site. Our work indicates that *Bd* is present in anuran communities in lowland Panamá, including the Darién province, and that the intensity of the infection may vary among species from different habitats and with different life histories. The population-level consequences of *Bd* infection in amphibian communities from the lowlands remain to be determined. Detailed studies of amphibian species from the lowlands will be essential to determine the reason why these species are persisting despite the presence of the pathogen.

## Introduction

Habitat loss and overexploitation are two of the main causes of biodiversity loss on the planet [1]. Amphibians are also threatened by chytridiomycosis [2], [3], an infectious disease caused by the chytrid fungus *Batrachochytrium dendrobatidis* (*Bd*). In recent decades, more than 40% of amphibian species have become vulnerable to extinction [1], and *Bd* has been detected in at least 48% of the amphibian species studied worldwide [4], [5]. In the tropics, drastic amphibian declines in forested protected areas have been clearly associated with chytridiomycosis [6], [7].

In the Neotropics, dramatic amphibian declines associated with *Bd* infection have been extensively documented in highland forests [6], [8], [9], [10] where the greatest losses in species diversity and population abundance in response to establishment of the pathogen have been described [11]. Moreover, a wave of infection spreading from Mexico down through Central America to Panamá has been thoroughly described [12], [13], [14]. In addition, *Bd* infection has been spreading across highland forests in South America including the Colombian Andes [15], [16], [17]. Thus, the Darién region of Panamá and Colombia is considered one of the last *Bd* naïve areas in Central America. Although invasion of *Bd* was thought to be inevitable, no surveys before this study have confirmed the presence of the pathogen into this region. However, in 2010, two frogs out of 93 individuals were infected with *Bd* in Tortí, a site at the Panamá Province close to the Darién [18].

*Bd* has been detected in lowland forests [10], [13], [19]–[22]; however, in low elevation sites there is little evidence of population declines associated with *Bd* [20]. One of the possible reasons for the absence of dramatic declines in the lowlands is that environmental conditions, such as temperature and moisture, are not optimal for *Bd* growth and successful colonization [23], [24], [25]. In addition, it is possible that lowland species are less susceptible to infection because of physiological and ecological traits, such as differential immune response, production of antimicrobial peptides, presence of symbiotic beneficial bacterial, behavioral patterns and habitat associations [19], [26]–[29]. Overall, the study of amphibian species persisting in the lowlands with *Bd* is a key component to understanding the nature of the disease, as well as how this pathogen spreads and colonizes new areas and hosts.

In this study, we determined the prevalence and intensity of *Bd* infection in three species of frogs from the tropical forests of Panamá. These amphibian species, *Agalychnis callidryas, Dendropsophus ebraccatus,* and *Craugastor fitzingeri*, are common in the lowlands, although their habitat distribution reaches highland forest up to 820–1520 m [30], [31]. *A. callidryas* and *D. ebraccatus* are nocturnal treefrogs (family: Hylidae) that spend most of the time in the forest canopy except for the breeding season, when individuals can be found on low vegetation near ponds; whereas *C. fitzingeri* (family: Craugastoridae) is mainly a nocturnal and terrestrial species usually found in the leaf litter and also along the margins of streams. The two treefrogs are pond breeders with arboreal eggs and aquatic larvae, whereas *C. fitzingeri* is a direct developer.

Previous studies have determined that *Bd* prevalence and infection intensity can vary between species from different habitats and with different life histories. For instance, *Bd* prevalence tends to be higher in frogs from riparian habitats than in frogs from terrestrial habitats [19], and breeding habitats can be important predictors of infection intensity [22], [32], [33]. Based on the contrasting life histories, we hypothesized that *Bd* infection, if present, could have different patterns among the three frog species that we studied. We propose two contrasting scenarios; in the first *C. fitzingeri* may have less exposure to *Bd* since all life stages of this frog are terrestrial, whereas the treefrogs *A. callidryas* and *D. ebraccatus* have an aquatic tadpole stage during which they may have more exposure to the aquatic zoospores of *Bd* [28]. Therefore, we would expect *C. fitzingeri* to have lower prevalence and intensity of infection in contrast to the treefrogs. Alternatively, it is likely that the treefrogs’ habitat as adults (the forest canopy) is warmer and drier than the leaf litter that terrestrial frog species inhabit. If *Bd* infection occurs during the adult stage, *C. fitzingeri* could be more frequently exposed to the pathogen than *A. callidryas* and *D. ebraccatus* due to differences in habitat temperature and moisture levels [34].

We evaluated *Bd* prevalence and infection intensity in two areas where it had been previously detected in Panamá, including one lowland site and one montane site, but we also extended our survey to eastern lowland areas in Panamá ranging from the east side of the Panama canal to the Darién area. We hypothesized that *Bd* would still be present in montane areas with low prevalence and intensity values characteristic of an enzootic infection status [13], [20]. In addition, based on previous work on the presence of *Bd* across Panamá [12], [13], [18], we predicted that *Bd* would be also present in lowland areas in eastern Panamá.

## Methods

### Ethics Statement

Scientific collection permits were provided by the Panamanian authorities (Autoridad Nacional del Ambiente): permits SE/A-47-12, SEX/A-65-12, SEX/A-77-12, SEX/A-89-12. Animal care protocols were approved by the Smithsonian Tropical Research Institute’s Animal Care Commitee: protocol 2011-1110-2014 and by Virginia Tech’s Animal Care Committee: protocol 11-105-BIOL.

### Study Sites and Sample Collection

**Figure 1:**
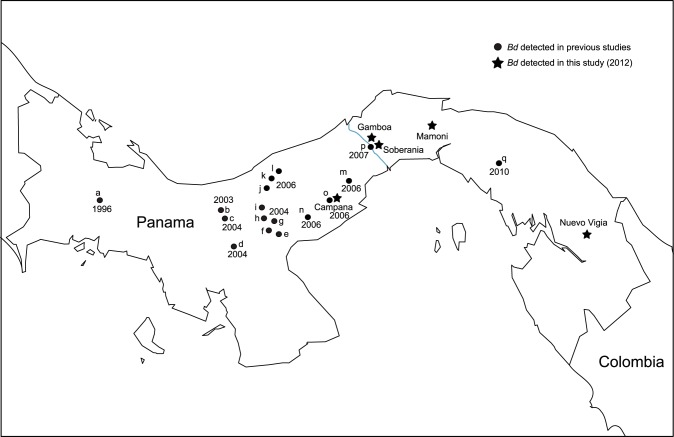
Map of Panamá showing the sites where *Bd* has been detected in previous studies and this study. The year next to each black circle shows the date when *Bd* was **first** detected on each site. **a** = Fortuna [8]; [50]. **b**, **c**, **d**, **e**, **f**, **g** and **h** = Parque Nacional Santa Fe, Altos de Piedra, Santiago marsh, Road pools, Río Grande watershed, Río Colorado watershed at bridge, Río Colorado watershed respectively [19]. **i** = El Copé [6]. **j**, **k**, **l** and **m** = La Rica, Palmarazo, Cuatro Callitas and Cerro Trinidad respectively [20]. **n** = El Valle [51]. **o** and **p**  =  Parque Nacional Altos de Campana and Parque Nacional Soberanía [13]. **q** = Tortí [18].

We sampled 203 individuals of the three species at one highland site (above 800 m) and four lowland sites throughout Panamá (Figure 1) during the rainy season (months of July, August and September 2012). The sites ranged from 29–845 m in elevation (Table 1). At the highland site (Parque Nacional Altos de Campana) and one lowland site (Parque Nacional Soberanía) *Bd* has previously been reported [13], [20]. At the three additional lowland sites (Gamboa, Mamoní and Nuevo Vigía/Icunatí), *Bd* has not been reported. *A. callidryas* and *D. ebraccatus* were sampled at all five sites while *C. fitzingeri* was only found at three of the five sites (Gamboa, Soberanía and Mamoní); sample sizes ranged from 7–35 individuals per species per site (Table 2).

**Table 1:** Description of the sites analyzed in this study.

Province	Site	Species sampled	Site description	Latitude (N)	Longitude (W)	Elevation (m)	Date when Bd was first detected
Panamá	Parque Nacional Altos de Campana	*A. callidryas* and *D. ebraccatus*	Vegetation near ephemeral pond	08^°^ 40.644 ′	079^°^ 55.72′	824	June 2006^a^
Panamá	Parque Nacional Soberanía	*A. callidryas* and *D. ebraccatus*	Vegetation near permanent pond	09^°^ 06.117′	079^°^ 40.928′	50	September 2007^a^
		*C. fitzingeri*	Leaf litter near stream	09^°^ 04.511′	079^°^ 39.537′	64	
Panamá	Gamboa	*A. callidryas* and *D. ebraccatus*	Vegetation near ephemeral pond	09^°^ 06.833′	079^°^ 41.798′	40	July 2012 (this study)
		*C. fitzingeri*	Leaf litter near Drainage ditch	09^°^ 06.946′	079^°^ 41.923′	47	
Panamá	Mamoní	*A. callidryas* and *D. ebraccatus*	Vegetation near ephemeral pond	09^°^ 18.382′	079^°^ 07.744′	191	July 2012 (this study)
		*C. fitzingeri*	Leaf litter near stream	09^°^ 19.231′	079^°^ 08.586′	245	
Darién	Nuevo Vigía and Icunatí	*A. callidryas* and *D. ebraccatus*	Vegetation near permanent pond	08^°^ 22.448′	077^°^ 46.075′	29	July 2012 (this study)
		*D. ebraccatus*	Vegetation near permanent pond	08^°^ 21.798′	077^°^ 42.637′	45	

**a  = ** Woodhams et al. [13].

**Table 2:** *Bd* Prevalence and infection intensity among three lowland species of frogs.

Site	Species	#Individuals (#Infected)	*Bd* Prevalence (CI 95%)^a^	*Bd* Zoospore equivalents (CI 95%)^b^
Parque Nacional Altos de Campana	*A. callidryas*	15 (4)	26.67 (10.89–51.95)	0.30 (0.09–0.63)
	*D. ebraccatus*	15 (4)	26.67 (10.89–51.95)	0.24 (0.07–0.41)
	**All species per site**	**30 (8)**	**26.67 (14.18–44.45)**	**0.27 (0.15–0.47)**
Parque Nacional Soberanía	*A. callidryas*	15 (3)	20 (7.04–45.18)	0.51 (0.09–0.89)
	*D. ebraccatus*	15 (0)	0 (0–20.38)	0 (−)
	*C. fitzingeri*	15 (6)	40 (19.82–64.25)	36.56 (3.76–129.3)
	**All species per site**	**45 (9)**	**20 (10.90–33.82)**	**24.54 (2.69–105.25)**
Gamboa	*A. callidryas*	15 (4)	26.67 (10.89–51.95)	0.47 (0.08–0.83)
	*D. ebraccatus*	15 (1)	6.67 (3.41–29.81)	0.06 (−)
	*C. fitzingeri*	15 (2)	13.33 (37.36–37.88)	0.06 (0.05–0.06)
	**All species per site**	**45 (7)**	**15.56 (7.74–28.78)**	**0.30 (0.04–0.51)**
Mamoní	*A. callidryas*	20 (5)	25 (11.19–46.87)	0.09 (0.07–0.14)
	*D. ebraccatus*	9 (3)	33.33 (12.05–64.57)	0.15 (0.05–0.22)
	*C. fitzingeri*	7 (1)	14.28 (7.31–51.31)	0.05 (−)
	**All species per site**	**36 (9)**	**25 (13.75–41.07)**	**0.11 (0.07–0.17)**
Nuevo Vigía and Icunatí	*A. callidryas*	12 (4)	33.33 (13.81–60.93)	0.14 (0.12–0.16)
	*D. ebraccatus*	35 (12)	34.28 (20.83–50.84)	0.18 (0.13–0.25)
	**All species per site**	**47 (16)**	**34.04 (22.16–48.32)**	**0.17 (0.16–0.42)**
	Total	**203 (49)**		

**CI 95%  = ** Confidence intervals based on 95% confidence.

**a = **Infection prevalence = % of infected individuals.

**b = **Average number of zoospore equivalents on infected individuals according to JEL423 standards.

Within each site, *A. callidryas* and *D. ebraccatus* were always found on vegetation surrounding ponds, where they come down from the canopy to breed. In contrast, *C. fitzingeri* was usually found in leaf litter close to streams. Frogs were collected at night and placed in sterile plastic bags until swabbing. Frogs were rinsed with 50 ml sterile distilled water to eliminate transient bacteria, which was important for a parallel study, and were then swabbed using a modified protocol from Hyatt et al. [35] that consisted of swabbing with a MW113 sterile rayon-tipped swab (Medical Wire & Equipment, Corsham, Wiltshire, England) 10 times back and forth on the ventral surface and 5 times in one direction on each thigh and rear foot for a total of 40 strokes. Skin swabs were kept at 4°C during fieldwork and once in the lab the swabs were immediately frozen and kept at −80°C until processing.

### DNA Extraction and *Bd* Detection

Whole genomic DNA was extracted from skin swabs using the DNeasy Blood and Tissue kit (Qiagen, Valencia CA, USA) according to the manufactureŕs instructions. The DNA was resuspended in a final volume of 100 µl.

Determination of *Bd* infection status was performed by Taqman real-time PCR assay according to Boyle et al. [36]. We tested for PCR inhibition and false-negatives through the use of internal positive controls using a subset of randomized samples as described previously [35]. Briefly, internal controls are synthetic amplicons whose sequence is not known to occur in nature (Applied Biosystems Exogenous Internal Positive Controls, Life Technologies, Carlsbad, CA). We performed multiplex PCR reactions that contained the template of our test samples detected by FAM labeled probes and the synthetic amplicon detected by VICTM labeled probes (TaqMan). In the absence of inhibitors, internal positive controls are amplified with the same efficiency in reactions that contained the tests samples as in reactions without them (negative control). No PCR inhibition was detected in our samples. DNA standards of *Bd* strain JEL423 were prepared as described previously [36]. The serial dilutions were prepared for 0.1–10,000 zoospore genome equivalents (from now on zoospore equivalents).

*Bd* strains have variable copy numbers as well as multiple haplotypes of the ITS1-5.8S DNA fragment [37]. Moreover, *Bd* strains have variable chromosomal copy numbers among strains and within strains [38], [39]. Based on the full diploid genome of JEL423 [40] this strain contains 22 identical copies of the ITS1-5.8S DNA fragment. In this study, we considered 22 copies as the minimum copy number that our reference strain JEL423 can have, however it is important to consider that this strain may have a higher number of copies due to changes in polyploidy and in the copy number of the ITS-5.8SRNA fragment that could have happened due to genomic rearrangements during passages in the laboratory [38; Longo A, pers. comm.]. Using the JEL423 genome as reference, one copy of the ITS1-5.8S DNA fragment equals 0.045 zoospore equivalents, and therefore values above this were considered positive for *Bd*. Three PCR reactions were done per each sample as described by Boyle et al. [36]. In addition, if one or more of the triplicate samples yielded a value between 0.045–1 zoospore equivalents, the qPCR was repeated in triplicate for a total of six reactions. Zoospore equivalents were obtained by averaging the replicated values per sample.

### Statistical Analyses

Prevalence values were calculated as the proportion of infected individuals per site per species. Infection intensity values (zoospore equivalents) per site per species were the mean values obtained from the samples that were infected.

For prevalence, 95% confidence intervals based on a binomial distribution were calculated using the Wilson Interval. For infection intensity, 95% confidence intervals (zoospore equivalents) were obtained using a bootstrap approach (BCa). Iterations of the bootstrap analyses ranged between 10,000–100,000 depending on the number of iterations that were needed for convergence. In the cases where groups were only composed of a single value, or when the value from two samples was the same, no confidence intervals could be calculated. Both confidence intervals (prevalence and infection intensity) were calculated as described by Kilburn et al. [20] using R version 2.15.2 (2012-10-26).

*Bd* prevalence (proportion of infected individuals) and infection intensity (*Bd* loads on infected individuals) were compared across sites within each species using a generalized linear model. Data were grouped in different categories according to species and site and analyzed with a single factor model. For prevalence we used a binomial distribution. For infection intensity we estimated that a lognormal model distribution best fit the data based on Akaike’s Information Criterion (AIC). For infection intensity, categories with only one value were not considered in the analysis. For each species, contrasts were constructed to test for differences in prevalence and infection intensity between sites. We also compared prevalence and infection intensity between species in the sites where all three species were present. For *A. callidryas* and *C. fitzingeri* at Gamboa and Soberanía, we tested whether differences in species’ responses depended on site. These analyses were conducted with PROC GLIMMIX using SAS (version 9.3). All statistical tests were conducted using 0.05 level of significance.

## Results

**Figure 2:**
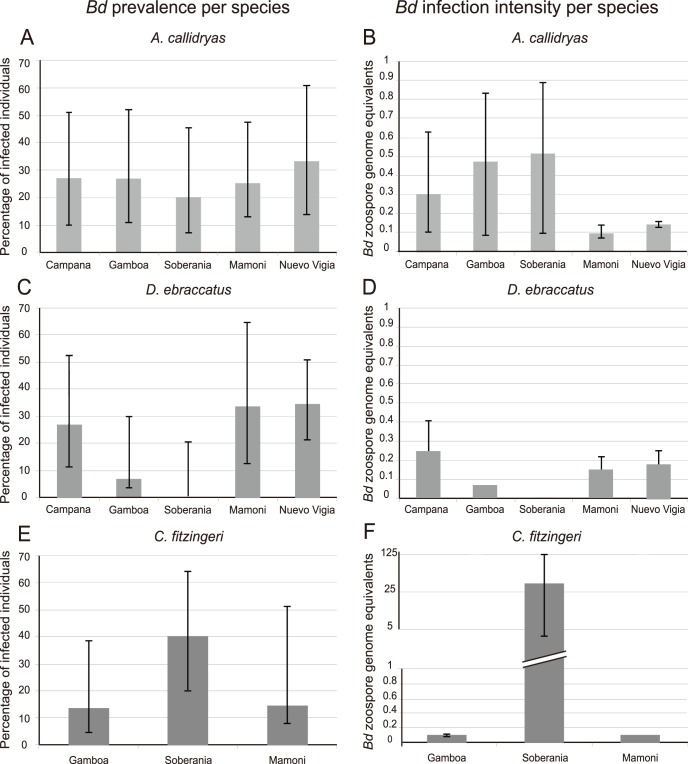
Mean *Bd* prevalence and infection intensity (zoospore genome equivalents) of three species of frogs across five sites in Panamá. NA =  No data available. Bars correspond to 95% confidence intervals. **A**, **C** and **E** show pathogen prevalence (% of infected individuals). **A)** *A. callidryas* across five sites **C)** *D. ebraccatus* across five sites. **E)** *C. fitzingeri* across three sites. **B**, **D** and **F** show Intensity of *Bd* infection based on the number of zoospore genome equivalents according to the reference strain JEL423. **B)** *A. callidryas* across five sites. **D)** *D. ebraccatus* across five sites. **F)** *C. fitzingeri* across three sites. Note that the scale of the Y axis begins as arithmetic and changes to logarithmic in order to present the data from the three sites on the same figure.

We found *Bd* infection at all five sites, including three sites in eastern Panamá that had not been sampled previously. Prevalence ranged from 15–34% per site (Table 2). Even though average prevalence varied across sites within each species (Figure 2A, C and E), no significant differences were found (F_(12,190)_ = 0.62_,_ p = 0.8272). Using contrasts, we tested for differences across sites within each species as well as for differences in prevalence between species in sites where the three species were present (Gamboa, Soberanía and Mamoní). None of these comparisons were significant.

We found that *Bd* infection intensity at all sites ranged from 0.11–24.54 zoospore equivalents per site (Table 2). Even though *Bd* prevalence was not significantly different across sites and species, overall infection intensity was significantly different (F_(9,37)_ = 6.27, p<0.0001). Using contrasts, we tested for differences across sites within each species. We found no significant differences across sites for either of the treefrog species (*A. callidryas* and *D. ebraccatus*; Figure 2B and D). However, infection intensity for *C. fitzingeri* differed between Gamboa and Soberanía (F_(1,37)_ = 21.77, p<0.0001; Figure 2F).

Infection intensity did not differ between *A. callidryas and D. ebraccatus (*F_(1,37)_ = 0.01, p = 0.9317) at sites where both species were present. Since *C. fitzingeri* was only present at three sites and categories with only one value were not considered in the analysis, we only compared infection intensity of this species with *A. callidryas* at Gamboa and Soberanía. Our results show that differences in *Bd* infection intensity between *A. callidryas* and *C. fitzingeri* depended on whether they were located at Soberanía and Gamboa, and this was reflected by a significant species-site interaction contrast (F_(1,37)_ = 11.83, p = 0.0015).

In sum, our results show that *Bd* is present in the lowland areas in eastern Panamá. Moreover the comparison between *A. callidryas, D. ebraccatus* and *C. fitzingeri* shows that infection intensity may vary among amphibian species at one lowland site.

## Discussion

In this study, we found *Bd* in areas previously known to have infected individuals, as well as in lowland sites presumed to be naïve to the pathogen. Our results, in combination with previous studies [10], [13], [18], [19], [20], [22] indicate that *Bd* is present in lowland tropical forests throughout Central America. Prevalence values obtained are similar to estimates obtained in previous studies from low elevation sites [20], [21], [22]. Furthermore, the prevalence at the only montane site analyzed in this study (Campana), was slightly higher than in the last report from that site [20] but similar to reports prior to 2010 [13].

One hypothesis to explain the presence of the fungus in far eastern Panamá is that *Bd* has continued spreading from Mexico to eastern Central America according to previous reports [12], [14]. In particular, Woodhams et al. [13] predicted that the fungus would spread to eastern Panamá and cause amphibian declines in this area by 2012. The results obtained in this study and the recent report in Tortí [18] are consistent with the prediction that *Bd* would continue to spread; however, no reports of amphibian declines from these regions have been published. An alternative hypothesis is that *Bd* may also be spreading in eastern Panamá from Colombia, since additional spreading waves have been documented in South America [12], [17]. This work is the first to test for and detect the presence of *Bd* in the Darién province. To our knowledge there are no previous reports indicating the absence of the fungus prior to 2012; therefore we cannot conclusively test the hypothesis that *Bd* has spread to this region in eastern Panamá. Furthermore, distinguishing the source of *Bd* in eastern Panamá might be feasible through the use of additional molecular markers such as microsatellite loci [41].

Current information about *Bd* infection and its effects on anuran diversity and abundance in lowland tropical forests is relatively scarce and inconclusive. Previous work in the lowlands in western Panamá described sick individuals with *Bd* infections and also reported lower species richness in the anuran communities once *Bd* was established [20]. In contrast, a study of anuran communities at Soberanía, east of the canal, did not show any signs of declines or changes in species richness, even though *Bd* was present at this site [13]. Determining the causes of amphibian declines in the lowland tropical forest can be complex given that these areas are often severely impacted by human activities such as farming and ranching. Moreover, there may be interactions between these different causes of amphibian declines. For example, infection intensity and prevalence of *Bd* in perturbed areas can be lower than in natural environments that are not impacted by anthropogenic activities such as deforestation [42]. To distinguish between all these factors additional studies that relate the arrival and presence of *Bd* to population declines and changes in community structure are necessary in lowland sites.

Previous studies in the Neotropics have focused on studying *Bd* infection in all or the majority of the species present in a site [6], [10], [13], [15], [19], [20]. Instead, we focused on obtaining larger samples of three species that are abundant in the lowlands to test for differences in pathogen infection levels. In this respect, our study shows that infection levels on *C. fitzingeri* can be significantly higher than *A. callidryas* and *D. ebraccatus* in sites like Soberanía. These three species persist in infected sites in both the highlands and lowlands of Panamá (Hughey and Ibañez, pers. obs.); however, differences in their life history and behavior may play a major role in the way they contend with chytridiomycosis. Evidence for differential patterns of *Bd* infection has been previously shown in amphibian species with different habitats and breeding behaviors [19], [22], [32], [33]. Treefrogs like *A. callidryas* and *D. ebraccatus* live in a very different habitat in comparison with terrestrial species such as *C. fitzingeri*. *Bd* has previously been detected in *C. fitzingeri* and other leaf litter dwellers from the *Craugastoridae* and *Dendrobatidae* families occurring in the lowlands, and therefore it has been suggested that *Bd* could be present in the moist forest floor [10], [18], [43]. Moreover, environmental variation, such as temperature and moisture, in local habitats (terrestrial versus canopy habitats) may play a major role in the persistence and colonization capacity of the pathogen [22], [34], [44], as well as in the potential host response to *Bd* exposure [45]. In addition, environmental fluctuations throughout seasons are also important factors involved in the fungal disease dynamics [22], [46]. The data shown here were obtained only during the breeding season (rainy season) and therefore our study does not address the infection prevalence and intensity fluctuations that might occur throughout the year. Collecting seasonal data for these species will be important to understand disease dynamics in the lowlands.

The infection intensity of *Bd* found on *C. fitzingeri* obtained in Soberanía was higher than the ones obtained in other lowland sites like Gamboa and Mamoní. One possible explanation for these results is that Soberanía is the most protected site, in contrast to the other lowland sites that are more impacted by anthropogenic factors. Disturbed environments, such as many lowland tropical forests, appear to be less suitable for maintaining high *Bd* prevalence and infection intensity due to microclimatic changes associated with vegetation loss [42], [44]. In relation to the two hypotheses presented in the Introduction, it appears that the leaf litter habitat is associated with higher infection intensities than the canopy habitat, although additional studies are needed to determine if this pattern is general.

Prior to this work, *Bd* loads have been analyzed with respect to zoospore equivalents, and individual amphibians were considered *Bd* negative if they had a zoospore equivalent below one. However, the genome of *Bd* is extremely complex and dynamic [37], [38], [39], and therefore, a zoospore (or single cell) may have multiple copies of the ITS-5.8SRNA fragment. Based on this evidence it is important to consider the number of copies that are present in reference strains used to quantify *Bd* loads [37]. Thus, the presence of a single copy in a sample can indicate the presence of *Bd* even if the zoospore equivalents are below one. Using this approach can avoid the underestimation of *Bd* loads in the wild.

The drivers of *Bd* disease dynamics in the lowlands are still an unknown and understudied subject. Even when *Bd* has been detected in the lowlands, zoospore loads have never been documented at the high levels seen in frogs from the highlands. For example, the last report of *Bd* in Panamá found mean infection intensities around 1–2×10^3^ zoospore equivalents in high elevation sites (890–1215 m), whereas low elevation sites (45–540 m) had mean intensity values between 40–70 zoospore equivalents [20]. As suggested previously, temperatures in the lowlands may not be optimal for the development of the disease [3], [47]. Furthermore, amphibian species from the highlands may be more susceptible than the ones in the lowlands. Susceptibility is dependent upon several factors including differential innate immune response, metabolic activity, production of antimicrobial peptides, presence of beneficial microbes on the skin and the ability to behaviorally thermoregulate [27], [29], [45], [48], [49]. In this respect we hypothesize that amphibian species from the lowlands are able to contend with infection in a way that the fungus rarely reaches lethal levels of zoospores on the amphibian skin. In addition, we hypothesize that *Bd* does not grow and reproduce as well under lowland conditions and that this allows amphibian defenses to be more effective. The study of anuran populations that have persisted in the lowlands despite the presence of *Bd* will be fundamental for understanding the mechanisms by which these species are able to survive the disease. This knowledge may allow us to develop conservation strategies to prevent future declines and extinctions of susceptible amphibian species in the tropics.
